# Structural and functional studies of the HIV-1 pre-integration complex

**DOI:** 10.1186/1742-4690-10-S1-P76

**Published:** 2013-09-19

**Authors:** Nicolas Levy, Sylvia Eiler, Karine Pradeau-Aubreton, Corinne Crucifix, Aurélie Schaetzel, Robert Drillien, Vincent Parissi, Stéphane Emiliani, Yves Mely, Patrick Schultz, Marc Ruff

**Affiliations:** 1 Institut de Génétique et de Biologie Moléculaire et Cellulaire, UDS, U596 INSERM, UMR7104 CNRS, Illkirch, France; 2 Laboratoire de Microbiologie Fondamentale et Pathogénicité, CNRS (UMR5234), Université de Bordeaux 2, Bordeaux, France; 3 Institut Cochin, Université Paris Descartes, CNRS (UMR8104), INSERM (U567), Paris, France; 4 Laboratoire de Biophotonique et Pharmacologie. UMR 7213 CNRS, UDS, Faculté de Pharmacie, Illkirch, France

## Background

To replicate their genome retroviruses need two key enzymes, the viral reverse transcriptase (RT) and integrase (IN). Soon after viral infection of a target cell, the genomic RNA is reverse transcribed by RT to generate the double-stranded viral DNA that interacts with viral and cellular proteins to form the pre-integration complex (PIC). IN is a key component of the PIC and is involved in several steps of retrovirus replication notably in reverse transcription, nuclear import, chromatin targeting and integration. IN cannot perform these functions on its own and need to recruit host cell proteins to efficiently carry out the different processes. Retroviral INs are proteins showing high inter-domain flexibility which accounts for their ability to interact with multiple partners, which in turn chaperone PIC formation and its multiple functions in integration. We assume that the different functions of the PIC in viral infection reflect the different conformations of their protein components as well as post-translational modifications. Biochemical and structural studies of the PIC have long been hampered due to the dynamics of composition and the intrinsic flexibility its components such as IN. We demonstrated that the low solubility and inter-domain flexibility can be circumvented by forming stable and specific complexes with DNA or protein co-factors and by post-translational modifications.

## Material and methods

Hundreds of milligrams of stable complexes are needed for *in vitro* functional and structural studies. For this, we develop new technologies for high molecular weight transient complexes production as well as for functional and structural analysis (mammalian cell system for protein production, *in vitro* functional analysis and complexes characterization). Structures have been solved by cryo-electron microscopy (Cryo-EM) and atomic model structures were fitted in the low resolution map using normal mode flexible fitting.

## Results

We reconstruct *in vitro* stable and soluble complexes around IN, the core protein of the PIC. We solved by cryo-EM the structures of the IN/LEDGF/DNA (Michel e*t al*, 2009) and IN/LEDGF/INI1/DNA (Maillot *et al*, 2013) complexes. Structures together with functional assays gave important hints on the functional role of LEDGF and INI1 in HIV-1 infection. Other sub-complexes of the PIC have been characterized in our lab. One of them, the IN/transportin-SR2/VBP1 complex is involved in the PIC nuclear translocation (cryo-EM structure in progress).

## Conclusions

Our results suggest that the function of INI1 (the core protein of the swi/snf chromatin remodeling complex) in HIV-1 replication is to stabilize the PIC in the host cell, by maintaining integrase in a stable constrained conformation which prevents nonspecific interactions and auto integration on the route to its integration site within nucleosomes, while LEDGF (a transcriptional co-activator) organizes and stabilizes an active integrase tetramer suitable for specific vDNA integration.

